# Spatial Analysis of Anthropogenic Landscape Disturbance and Buruli Ulcer Disease in Benin

**DOI:** 10.1371/journal.pntd.0004123

**Published:** 2015-10-16

**Authors:** Lindsay P. Campbell, Andrew O. Finley, M. Eric Benbow, Jenni Gronseth, Pamela Small, Roch Christian Johnson, Ghislain E. Sopoh, Richard M. Merritt, Heather Williamson, Jiaguo Qi

**Affiliations:** 1 Department of Ecology and Evolutionary Biology, University of Kansas, Lawrence, Kansas, United States of America; 2 Biodiversity Institute, University of Kansas, Lawrence, Kansas, United States of America; 3 Department of Forestry, Michigan State University, East Lansing, Michigan, United States of America; 4 Department of Geography, Michigan State University, East Lansing, Michigan, United States of America; 5 Department of Entomology, University of Michigan State University, East Lansing, Michigan, United States of America; 6 Department of Osteopathic Medical Specialties, Michigan State University, East Lansing, Michigan, United States of America; 7 Center for Global Change and Earth Observations, Michigan State University, East Lansing, Michigan, United States of America; 8 Department of Microbiology, University of Tennessee, Knoxville, Tennessee, United States of America; 9 Programme National de Lutte contre la Lèpre et l’ulcère de Buruli, Cotonou, Benin; 10 Institut régional de santé publique, université d'Abomey-calavi, Ouidah, Bénin; 11 Department of Biological Sciences, Mississippi State University, Starkville, Mississippi, United States of America; National Institute of Parasitic Diseases China CDC, CHINA

## Abstract

**Background:**

Land use and land cover (LULC) change is one anthropogenic disturbance linked to infectious disease emergence. Current research has focused largely on wildlife and vector-borne zoonotic diseases, neglecting to investigate landscape disturbance and environmental bacterial infections. One example is Buruli ulcer (BU) disease, a necrotizing skin disease caused by the environmental pathogen *Mycobacterium ulcerans* (MU). Empirical and anecdotal observations have linked BU incidence to landscape disturbance, but potential relationships have not been quantified as they relate to land cover configurations.

**Methodology/Principal Findings:**

A landscape ecological approach utilizing Bayesian hierarchical models with spatial random effects was used to test study hypotheses that land cover configurations indicative of anthropogenic disturbance were related to Buruli ulcer (BU) disease in southern Benin, and that a spatial structure existed for drivers of BU case distribution in the region. A final objective was to generate a continuous, risk map across the study region. Results suggested that villages surrounded by naturally shaped, or undisturbed rather than disturbed, wetland patches at a distance within 1200m were at a higher risk for BU, and study outcomes supported the hypothesis that a spatial structure exists for the drivers behind BU risk in the region. The risk surface corresponded to known BU endemicity in Benin and identified moderate risk areas within the boundary of Togo.

**Conclusions/Significance:**

This study was a first attempt to link land cover configurations representative of anthropogenic disturbances to BU prevalence. Study results identified several significant variables, including the presence of natural wetland areas, warranting future investigations into these factors at additional spatial and temporal scales. A major contribution of this study included the incorporation of a spatial modeling component that predicted BU rates to new locations without strong knowledge of environmental factors contributing to disease distribution.

## Author Summary

Changes in land and use and land cover can impact ecosystems in unexpected ways, including changes in habitat suitability for environmental pathogens. Several studies have investigated the impacts of human disturbance to the landscape and changes in the composition of vector, host, and reservoir species in an altered area, but few studies have linked these disturbances to environmental pathogens. Buruli ulcer disease is a neglected tropical disease caused by the environmental pathogen *Mycobacterium ulcerans*. This study investigated land cover patterns surrounding villages in southern Benin to identify relationships between disturbed landscapes and disease prevalence. The authors were also interested in whether the drivers of disease prevalence had a spatial structure that could provide clues to environmental characteristics important to disease presence. Results suggested that villages surrounded by natural, or undisturbed wetlands had higher BU rates, and there was, in fact, a spatial structure to the pattern of disease prevalence. The authors used these outcomes to create the first continuous, BU risk map across southern Benin and Togo, even though the exact drivers of disease transmission remain unknown. Predicting potential risk adequately provides valuable information toward prevention, while helping to target public health resources more efficiently.

## Introduction

Land use and land cover (LULC) change at multiple spatial and temporal scales is one anthropogenic disturbance linked to infectious disease emergence [1]. Anthropogenic activities with major impacts on LULC are land degradation, including agriculture intensification and water projects, urbanization, and deforestation [2]. These activities can lead to ecological edge effects that promote disease emergence [3]. Further, these activities generate new pathways through which humans can interact with previously undisturbed environments, resulting in closer proximities to potential vectors, reservoirs, and isolated pathogens [3–6].

Advances in geographic information systems (GIS), remote sensing technologies, spatial statistical methods and computational capacities facilitate observation and quantification of anthropogenic landscape disturbances, providing the tools necessary to link landscape characteristics to disease incidence and to predict disease transmission risk across landscapes and through time [7]. While current research has focused largely on wildlife and vector-borne zoonotic disease emergence [8,9], exploring linkages between anthropogenically-disturbed landscapes and human bacterial infections has been given less attention, even though recent findings suggest that disturbances may contribute to the spatial distribution of environmental bacteria that pose a human health risk [10]. Quantification of landscape patterns related to bacterial disease emergence is a central component to mapping transmission risk because ecological drivers behind these pathogens are often poorly understood.

One example of this phenomenon is Buruli ulcer (BU) disease, a necrotizing skin disease caused by the environmental pathogen *Mycobacterium ulcerans* (MU) [11,12]. MU produces mycolactone, an immunosuppressive agent responsible for ulcer formation. Although the ecological drivers behind MU growth in the environment remain a mystery, empirical and anecdotal linkages exist between dramatic increases in BU cases since the 1980s and anthropogenic landscape changes [13]. These disturbances include, but are not limited to, deforestation, habitat fragmentation, aquatic ecosystem disturbances from dam construction and agriculture irrigation, changing farming practices, and mining activities [12]. Although BU is not transferred between persons, the mode or modes of transmission has not been determined, and no vaccine exists [14]. Therefore, identifying landscape patterns linked to BU incidence will provide a powerful tool for surveillance and prevention while affording opportunities to learn more about the ecology of the disease system.

Several past BU studies investigated landscape features related to BU disease. Research in the Amansie West District of Ghana identified a correlation between disease incidence and proximity to soils enriched with arsenic in low-lying farmlands [15]. An additional study in the Amansie West district identified a relationship between mean arsenic levels in soil and the spatial distribution of BU cases and between increased proximities to gold mining sites and the spatial distribution of cases within the district [16]. Mantey et al. [17] investigated linkages between potential surface runoff and BU incidence in the Amansie West and Upper Denkyira West Districts of Ghana, finding that BU cases correlated positively with a low to moderate potential for surface runoff and that higher numbers of cases correlated with lower potential maximum soil water retention values. A country-wide study in Côte d’Ivoire found an association between BU incidence and closer proximities to irrigated rice fields and to artificial dams [18], while a study by Marion et al. [19] postulated an association between the construction of a large dam in the Bankim district of Cameroon and the geographic expansion of BU cases.

An additional study in the Ankonolinga health district of Cameroon investigated fine-scale patterns of BU incidence within the area, finding closer proximities to the Nyong River, disturbed forest area, and cultivated wetlands to be significant risk factors for the disease [20]. A country-wide study in Benin determined that villages at low elevations within drainage basins, with variable wetness patterns, and surrounded by forest had higher BU risk [21]. A recent study in Benin suggested an inverse relationship between BU incidence and elevation [22], while Williamson et al. (2012) found incidences to be lowest at elevations <25m or at higher elevations of 90-100m. Wagner et al. [23] determined that BU incidence increased for villages surrounded by agriculture land and decreased with surrounding urban land use at broad scales (e.g., 20 km radius around a village).

While offering important insight into linkages between landscape features and BU incidence, these studies focused on landscape composition rather than its configuration, neglecting landscape patterns as overall indicators of disturbance. Past studies using landscape metrics suggested that land cover patches with more uniform shapes (e.g. corresponding to roadways or managed forest patches) are indicators of potential anthropogenic disturbance [24]. In contrast, land cover patches with more complex shapes, such as natural wetlands, often represent undisturbed landscapes [25]. In addition, higher numbers of patches can suggest potential habitat fragmentation [26].

Although several previous BU studies have utilized spatially-referenced data, only a fraction of studies investigating landscape and environmental variables incorporated spatial statistical modeling approaches into their analyses [16,20,21,23]. Tobler’s first law of geography states that “everything is related to everything else, but near things are more related than distant things” [27], a verbal statement of the concept of spatial autocorrelation. If a non-spatial regression model is used to investigate BU-environmental relationships, which is often the case, there is a high propensity to violate basic model assumptions, such as independent and identically distributed model residuals [28]. Models that account explicitly for spatial autocorrelation offer several advantages over non-spatial models, including avoidance of incorrect inferences regarding regression coefficient significance, or Type I errors [29–31]. Further, addition of components that explicitly accommodate residual spatial dependence to the class of models considered, often improves model fit and predictive performance [32].

The purpose of this study was to quantify effects of potential anthropogenic landscape disturbances on BU incidence in Benin, West Africa, using landscape metric calculations and spatial modeling approaches. We hypothesize (1) that land cover patches with configurations indicative of anthropogenic landscape disturbance surround villages with higher BU rates in southern Benin and (2) that a spatial pattern exists for drivers of BU incidence in Benin. Our final objective was to create a BU risk surface across southern Benin and southern Togo.

## Materials and Methods

**Fig 1:**
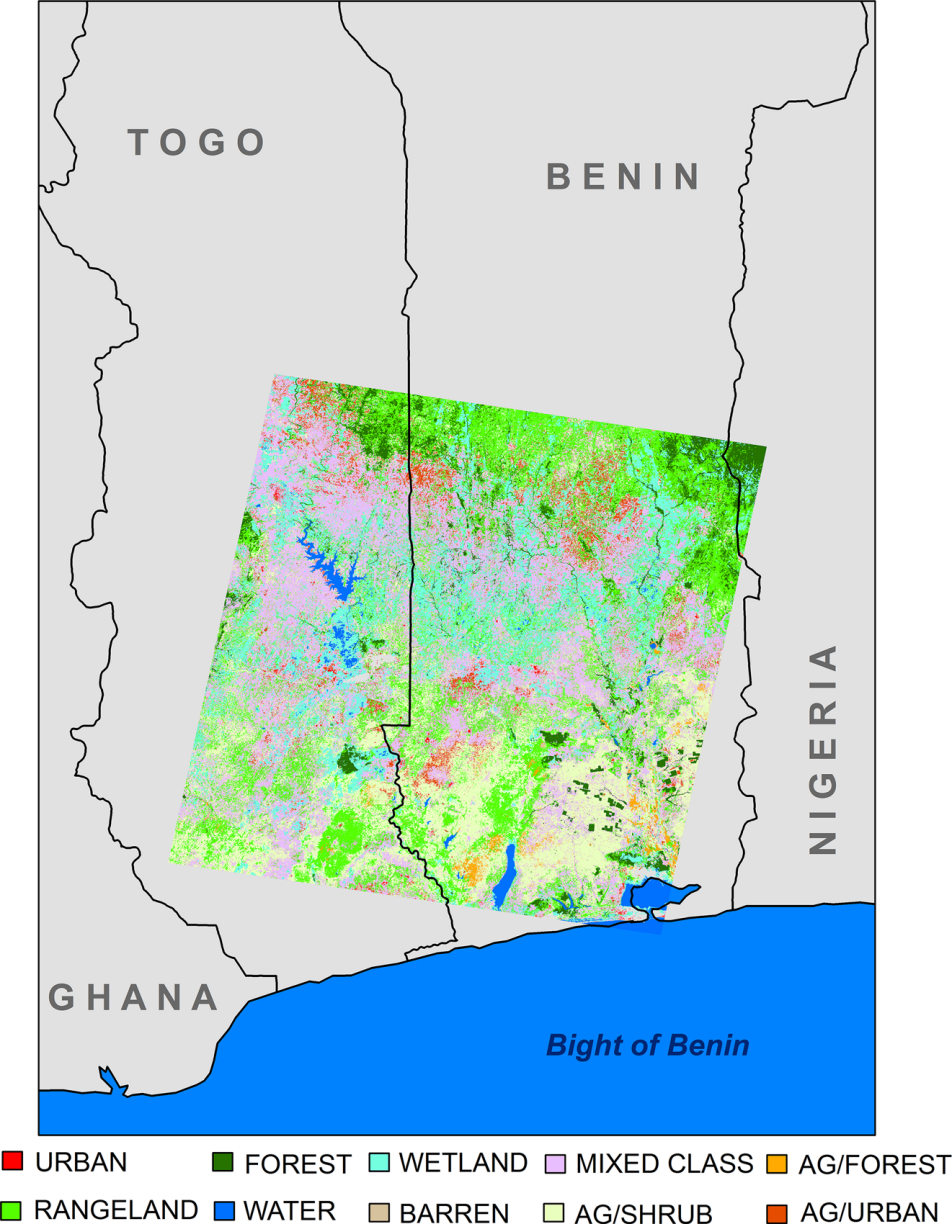
Study area and land cover classes.

The southern portions of Benin and Togo, West Africa comprise our study area, 6.30°N—8.17°N and 0.84°E to 2.48°E (Fig 1). Four major rivers flow through the study area, including the Couffu, Ouémé and Zou rivers in Benin, and the Mono River that delineates the southern border between Togo and Benin. BU is endemic in Benin and Togo, but incidence data from Togo were incomplete. Therefore, this study used BU case observations and corresponding environmental data in Benin to identify significant drivers, and then predicts BU risk across southern Benin and Togo.

Benin’s landscape consists primarily of woodland and shrub savannas, intermixed with cultivated areas and fallow fields, with semi-deciduous forests present in the southern region of the country [33]. Deforestation and environmental degradation are ongoing problems in the country, and the landscape continues to change rapidly. Food-crop and cotton cultivation expanded by 265% and 79% between 1986 and 1997, while firewood extraction and charcoal production contributed to a 30,000 ha/yr deforestation rate [33].

### BU Case Data

**Fig 2:**
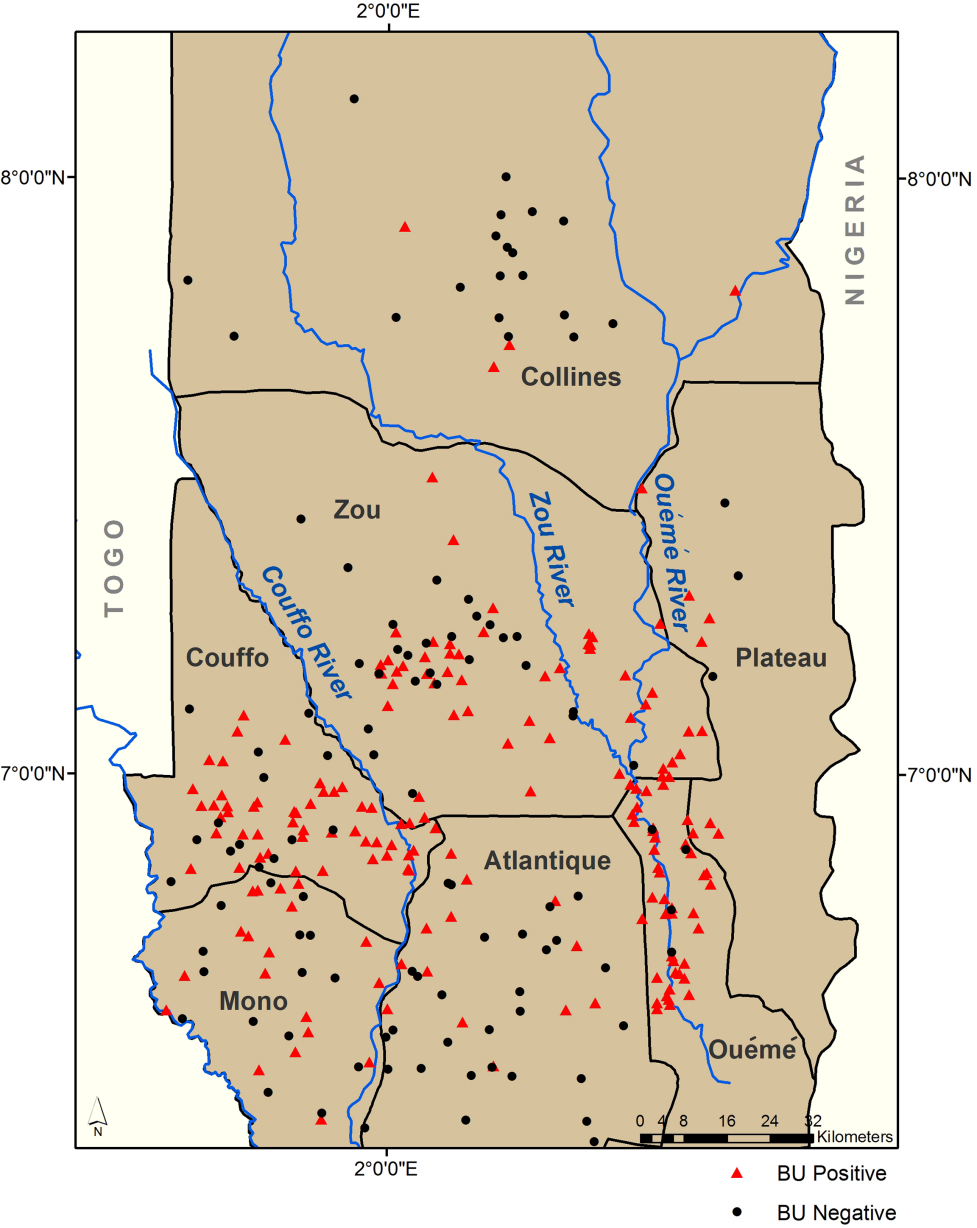
BU positive and BU negative community locations.

The Programme National de Lutte contre la Lèpre et l’ulcère de Buruli (PNLLUB) in Benin provided a subset of BU positive and BU negative villages in 2004 and 2005 for this analysis. A village was identified as BU positive if at least one case occurred there in 2004 or 2005. These data were obtained from World Health Organization (WHO) BU02 standardized forms, created using a community-based reporting system developed by the WHO to facilitate case reporting across geographic regions [34]. BU case counts, population, and latitude and longitude coordinates were provided for each village in the data set. A total of 292 villages, 183 positive and 109 negative, fell within the study area; 558 individual cases occurred, ranging between 1–29 cases per village (Fig 2). Data deposited in the Dryad repository [http://dx.doi.org/10.5061/dryad.j512f21p][35].

### Land Use and Land Cover Data

A land use and land cover (LULC) classification for southern Benin and Togo was performed using 30m resolution Landsat ETM+ imagery from 13 December 2000. The imagery was geometrically rectified and projected (UTM Zone 31N) before performing an unsupervised classification [36]; assignment of land cover classes followed Anderson’s Level I classification scheme [37], with the exception of four mixed classes. The final classification consisted of 10 classes total, including three mixed agriculture classes and a general mixed-use class that included pixels classified as belonging to >2 categories. Execution of a 5x5 statistical majority filter helped to eliminate classification noise [38]. Visual interpretation methods were used to delineate land cover classes [39–41] across the study area for a lack of ground truth data. Aggregated land cover classes were intended to help mitigate classification error. Data deposited in the Dryad repository [http://dx.doi.org/10.5061/dryad.j512f21p][35].

Forest, wetland, and mixed agriculture/forest classes were selected for this analysis based on potential linkages identified from empirical and anecdotal scenarios. Forest and wetland classes exhibited unique spectral signatures, with wetland signatures showing a lower spectral reflectance curve, particularly in the Near Infra-Red (NIR) range of the electromagnetic spectrum, while forest signatures demonstrated a sharper increase in the NIR range of the electromagnetic spectrum [42]. We noted larger uncertainty in separating the mixed agriculture and forest class from other non-forested classes because of the spatial resolution of our data.

### Landscape Metrics

Quantifying landscape patterns within concentric polygons is a common approach in multiscalar landscape analyses [43,44]. Concentric polygons with radii of 800m, 1200m, 1600m, and 2000m intervals from village centers acted as buffers within which land cover data were obtained for pattern analysis. These radii were chosen to characterize the landscape within distances traversed regularly by village residents, while maintaining extents large enough to quantify landscape patterns.

Initial calculation of a suite of landscape metrics quantified study class configurations within the concentric polygons using an 8-neighbor rule [45]. Of particular interest were landscape metrics that identify landscape disturbances linked to anthropogenic activities, for example fragmentation or uniform land cover patch shapes (refer to FragStats manual for greater detail [46]).

**Fig 3:**
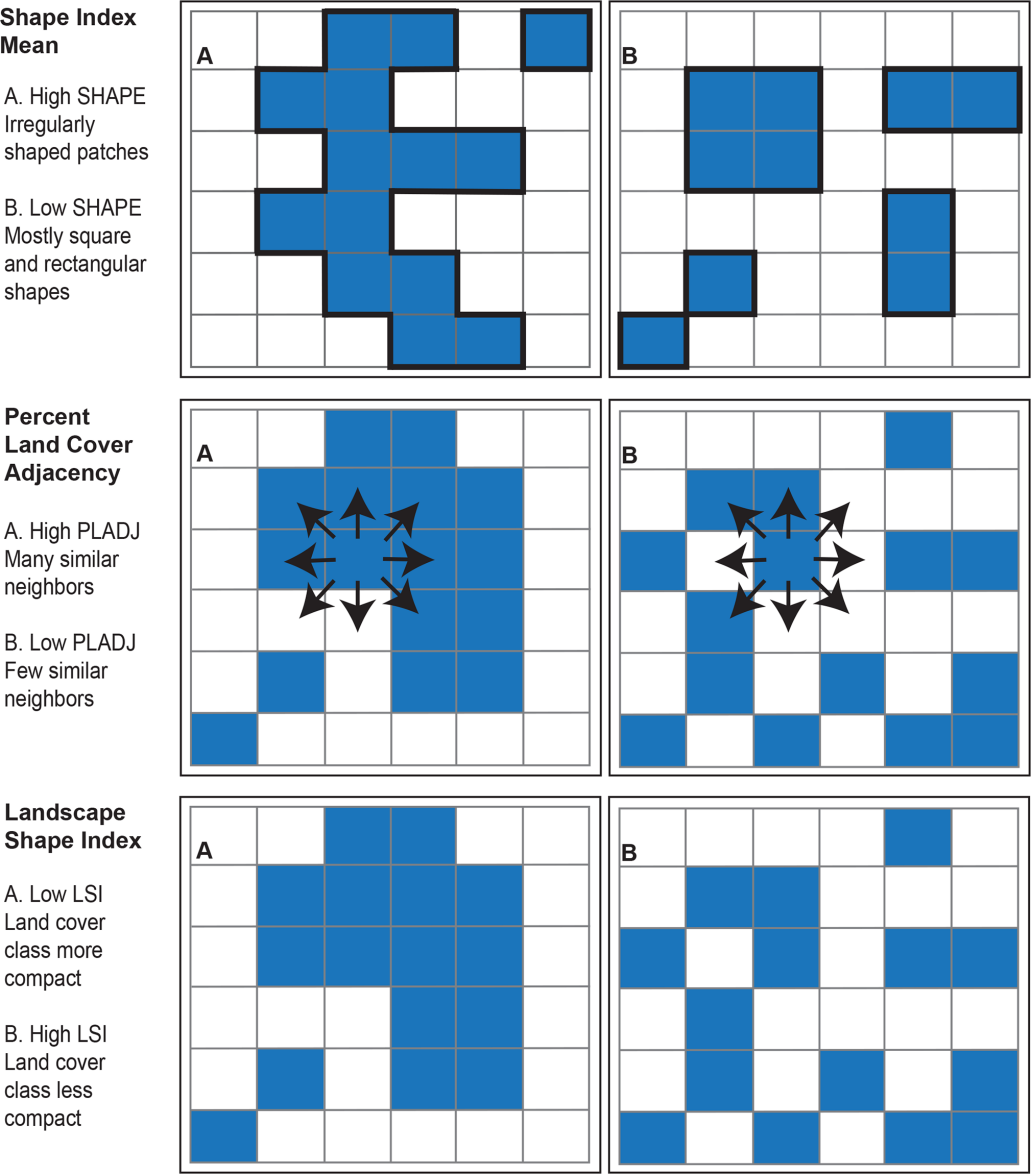
Examples of land cover configurations resulting in high and low values for the three landscape metric calculations.

Collinearity among landscape metrics is common (46). In a regression context, collinearity, or correlation, among predictor variables can cause problems with inference. Hence, applied regression texts suggest avoiding correlation among predictors beyond 0.7, see, e.g., [47]. Here, we took a conservative cut-off of 0.6 to reduce issues arising from collinearity. Potential predictor pairs were assessed using Pearson's correlation coefficient and Spearman's rank order correlation. Both correlation metrics yielded comparable results. Using this criterion and exploratory analysis using the models detailed in subsequent sections, the predictor variables we consider in the subsequent analyses are: 1) shape index mean, 2) percent land cover adjacency, and 3) landscape shape index. Fig 3 provides an illustration of land cover configurations which give rise to high and low values of each metric. The following metric calculation descriptions were derived from the FragStats documentation directly. Shape index mean (SHAPE_MN) characterized patch shape complexity and was calculated as

$$SHAPE=\frac{p_{ij}}{\text{min}p_{ij}}$$

where *p*_*ij*_ = perimeter of patch *ij* in terms of number of cell surfaces and min *p*_*ij*_ = minimum perimeter of patch *ij* in terms of number of cell surfaces [46]. Values closer to 1.0 indicate more uniformly-shaped land cover patches with complexity increasing as values increase.

Percent land cover adjacency (PLADJ) measured aggregation between patches of a similar class and is calculated as

$$PLADJ=\left(\frac{g_{ij}}{\sum_{k=1}^{m}g_{ik}}\right)$$

where *g*_*ij*_ = the number of like adjacencies (joins) between pixels of patch type (class) *i* based on a double-count method, and *g*_*ik*_ = the number of adjacencies (joins) between pixels of patch types (classes) *i* and *k*, also based on a double-count method [46]. High PLADJ values represent a more aggregated land cover class, while low PLADJ values represent a more fragmented land cover class.

The landscape shape index (LSI) is another land cover patch aggregation measurement. LSI was calculated using

$$LSI=\frac{e_{i}}{\text{min}e_{i}}$$

where *e*_*i*_ = perimeter of class *i* in terms of number of cell surfaces, which includes all landscape boundary and background edge segments involving class *i*, and min *e*_*i*_ = minimum total length of edge (or perimeter) of class *i* in terms of number of cell surfaces [46].

### Non-Spatial Models

The initial non-spatial generalized linear model (GLM) considered the n = 292 villages and coinciding predictor variables indexed by location, *S* = {***s***_*i*_,…, ***s***}, where each ***s*** is a vector recording the longitude and latitude in UTM Zone 31N projection. At generic location ***s***, the response variable *y*(***s***) was taken as the number of BU cases reported there. At generic location *s*, the response variable *y*(*s*) was taken as the number of BU cases reported in each village of population size *N*(*s*). We assumed *y*(*s*) followed a Binomial distribution with *N*(*s*) observations in each village and probability *p*(*η*(*s*)) of BU incidence, i.e., *y*(*s*) ∼ *Binomial*(*N*(*s*),*p*(*η*(*s*))). A logistic link function was used to model the probability of BU, i.e, *p*(*η*(*s*)) = exp(*η*(*s*)) / (1+*η*(*s*)). For the non-spatial models, the regression term *η*(*s*) = *x*(*s*)’*β* where the vector *x*(*s*) comprises an intercept, village-specific predictor variables (i.e., SHAPE ME, PLADJ, and LSI), and associated regression parameters *β*. We used a stepwise approach to explore combinations of predictor variables within candidate models over the 800m, 1200m, 1600m, and 2000m distances from village centers and each of the three land cover classes. Candidate models consisted of variables with a correlation coefficient < 0.6. Model outcomes were compared using a Deviance Information Criterion approach (DIC) [48], and individual variables within candidate models were eliminated in a stepwise process. Final models consisted of those with the lowest DIC values.

### Spatial Models

The non-spatial binomial GLMs assumed that model residuals were independent and identically distributed across the study domain [28], i.e., the included predictor variables capture any spatial patterns in the probability of BU cases. While this approach is adequate in the absence of spatial autocorrelation in model residuals, this assumption will often be unrealistic given the spatial structure of the observations [49]. As noted above, violations of this model assumption can result in misleading inference regarding the importance of predictors and subsequently erroneous predictions at locations where BU rates were not observed. To mitigate this issue, the non-spatial model was modified to allow for a spatially-varying intercept, via the addition of spatially structured random effects. Mapping of the random effects are often useful for identifying missing or unobserved predictor variables [50], in addition to accommodating any lurking residual spatial dependence, improving inference about the importance of the predictor variables, and increasing predictive performance.

The spatially varying intercept is included in the model by adding a spatial random effect, *w(* ***s*** *)*, to *η(* ***s*** *)*, *i*.*e*. *η*(***s***) = ***x*** *(* ***s*** *)’* ***β*** *+w(* ***s*** *)* where ***x*** *(* ***s*** *)* and ***β*** were defined previously. The spatial random effect was specified as a mean zero Gaussian Process with covariance function *C*(***s***_***1***_ **, *s***_***2***_ ***;θ*** *) = σ* ^*2*^ *ρ*(***s***_***1***_ **, *s***_***2***_ ***;*** *ϕ*), with ***s***_***1***_ and ***s***_***2***_ representing any two arbitrary locations and *σ* ^*2*^ is the process variance. We assumed an exponential spatial correlation function, *ρ*(·**,**·***;*** *ϕ*) = *exp*(*-ϕ||* ***s***_***1***_ − ***s***_***2***_ *||*), where *ϕ* controls the rate of spatial decay and *||* ***s***_***1***_ − ***s***_***2***_ *||* is the Euclidean distance between the locations [50]. See [51–53] for examples of spatial random effects applications in disease ecology research.

The model specification is completed by assigning prior distributions to all parameters. As customary, the regression coefficients ***β*** were assigned a multivariate Gaussian prior, ***β~N(μ*, *Σ)***, with ***μ*** set to a vector of zeros, and the matrix ***Σ*** specified with diagonal elements equal to 100 and off-diagonal elements to zero. Exploratory data analysis (EDA) using other diagonal variance values, i.e., 1000 and 10000, were assessed for the non-spatial and spatial models with results showing negligible impact of the prior choice on width or centering of posterior distributions.

The spatial variance component *σ* ^*2*^ was assigned an inverse gamma prior *IG(a*, *b)*, with the shape hyperparameter, *a*, set to 2 and the scale, *b*, parameter varied from 0.1 to 5 to assess the influence of the prior specification. Note, that following the *IG* definition in [54], with α = 2 the distribution mean is *b* and has infinite variance. EDA using the various specifications of *b* showed little influence on the posterior inference. Results presented in subsequent sections used an *IG*(2,1) prior for σ^2^. The process correlation parameter, *ϕ*, was assigned an informative prior (e.g., uniform over a finite range) with support across the maximum intersite distance among any two locations.

Model parameter distributions were estimated using Markov chain Monte Carlo (MCMC) methods employing an adaptive Metropolis (AM) algorithm with a 43% acceptance rate [55]. All models were generated using the spGLM function in spBayes R package [56]. MCMC chain starting values were obtained from the non-spatial models and subsequent posterior inference was based on 3 chains at 100,000 iterations measured over the four spatial extents (e.g. 800m, 1200m, 1600m, and 2000m).

Chain convergence was diagnosed using the Gelman-Rubin potential scale reduction factor [57]. Convergence for the non-spatial and spatial model parameters was typically achieved within 5,000–10,000 MCMC iterations. Parameter inference and subsequent predictions were based on post-convergence MCMC samples (i.e., burn-in was set at 10,000 iterations for all model parameters).

### Model Comparison and Verification

Models were compared using the popular Deviance Information Criterion (DIC). Like Akaike Information Criterion and similar model fit criteria used to compare fit among non-Bayesian models, lower DIC values indicate better performance [48]. To more fully assess predictive performance among the candidate models, the data sets were divided randomly into two subsets– 90% of the observations were used to fit the candidate models and 10% used to verify subsequent BU rate predictions. For each model, root mean squared prediction error (RMSPE) was calculated based on the 10% holdout set’s predicted and observed values, with lower values indicating improved expected performance [55].

### BU Risk Surface

**Fig 4:**
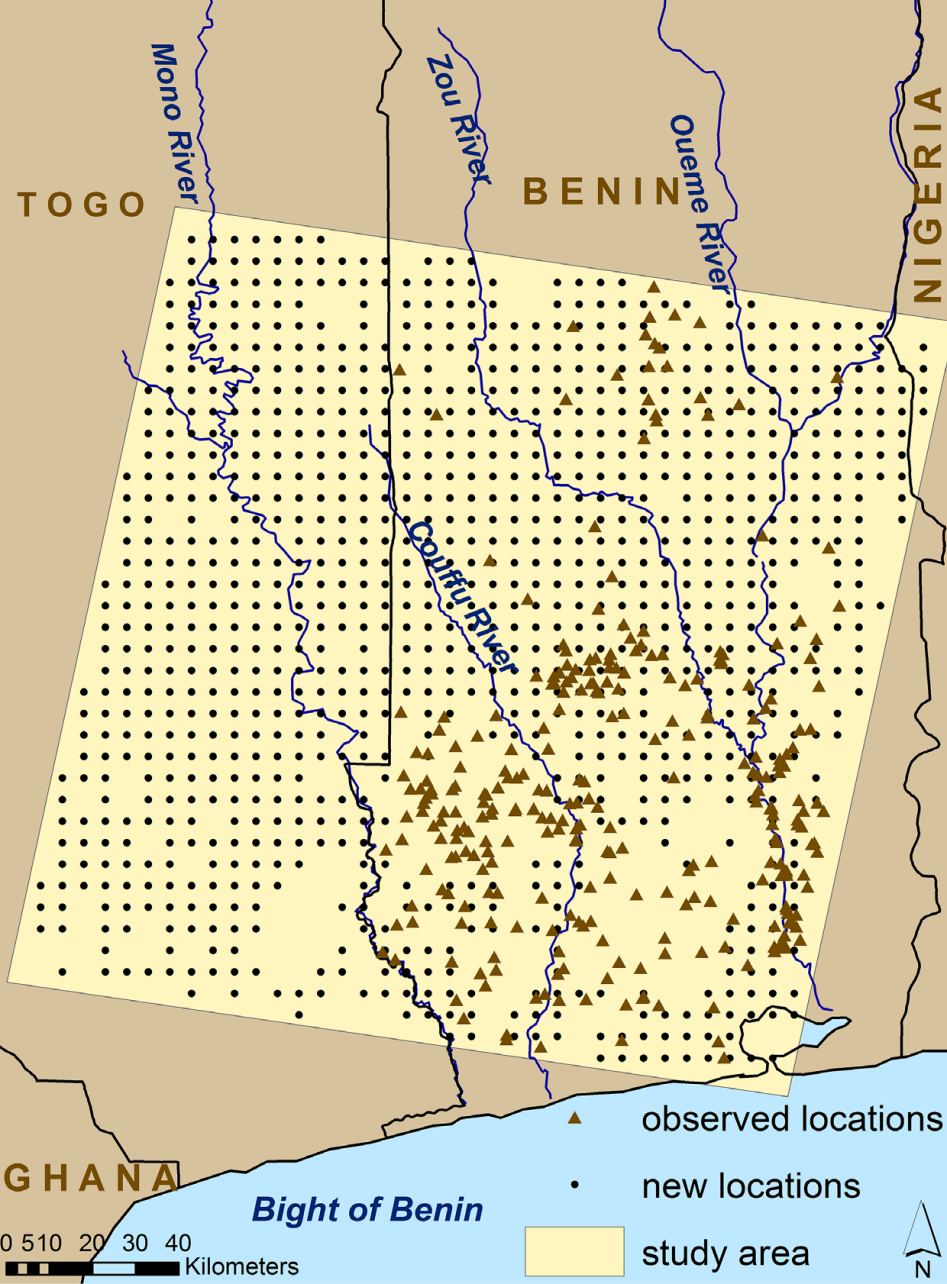
New locations gridded at 5 km intervals for BU rate predictions at unobserved locations.

We generated a total of 1029 new prediction locations in a gridded pattern at 5 km intervals, where BU rates were unobserved (Fig 4). Gaps among these location points represent areas where data were lacking and derivation of predictor variables was not possible for the “best” model class and distance interval. The predictive BU risk surface was created following Finley et al. (2008) and implemented in the spBayes R package. Here, the use of a Bayesian modeling paradigm is particularly advantageous because it provides access to the entire posterior predictive distribution at each new location [50], and hence, we can map any quantity of interest. For our purposes, these quantities include a map of grid cell specific posterior predictive distribution mean and variance.

## Results

The majority of BU positive communities in Benin were located in the southern region of the country (Fig 2). The departments of Zou and Couffo had the highest number of BU positive communities in our data set, with 51 and 59 locations. Ouémé, Mono, and Atlantique followed with 38, 19, and 14 BU positive communities, with Plateau and Collines having 8 and 4 positive locations from our sample data set.

### Spatial vs. Non-Spatial Models

The spatial GLMs produced lower DIC values than the non-spatial GLMs, suggesting that residual spatial dependence was present and that the spatial models achieved a better fit for every combination of predictor variables (Table 1). Comparison of regression coefficients between the spatial and non-spatial models shows that the non-spatial GLMs tend to overestimate the importance of the predictor variables—likely resulting from violation of the model assumptions related to non-correlated residuals (Table 2). For example, Forest was found to be significant at every spatial scale in the non-spatial model, while only 1200m Forest was significant in the spatial model. In addition, the percent land cover adjacency variable corresponding to the 2000m wetland class model reversed signs, again attributed to a violation of the basic model assumptions.

**Table 1:** Deviance Information Criterion for non-spatial vs. spatial models. Lower DIC values indicate a better model fit. Variable abbreviations correspond to metrics as follows: SHAPE_MN (shape index mean), PLADJ (percent land cover adjacency), LSI (landscape shape index).

*DIC Value*			
*Model*	*Variables*	*Non-Spatial*	*Spatial*
800 Forest	SHAPE_MN + PLADJ	1928	1780
800 Wetland	SHAPE_MN + PLADJ	3918	3855
800 Ag/Forest	SHAPE_MN + LSI	2000	1868
1_2k Forest	SHAPE_MN + LSI	3661	3288
1_2k Wetland	SHAPE_MN + LSI	5335	4779
1_2k Ag/Forest	SHAPE_MN + LSI	3902	3655
1_6k Forest	SHAPE_MN	3668	3258
1_6k Wetland	SHAPE_MN + LSI	5937	5223
1_6k Ag/Forest	SHAPE_MN + LSI	4345	4070
2k Forest	PLADJ	4394	3879
2k Wetland	PLADJ	6559	5889
2k Ag/Forest	SHAPE_MN + LSI	4712	4327

**Table 2:** Variable significance comparison of non-spatial versus spatial models. The “+” and “-”symbols indicate significant coefficients in the direction indicated by the symbol, and the “X” symbol indicates non-significant coefficients.

*Comparison of Variable Significance*			
Model	Variable	Non-Spatial Model	Spatial Model
800m Forest	SHAPE_MN	(+)	X
	PLADJ	(+)	X
800m Wetland	SHAPE_MN	(+)	X
800m Ag/Forest	SHAPE_MN	(+)	X
	LSI	(-)	(-)
1200m Forest	SHAPE_MN	(+)	(+)
	LSI	(-)	(-)
1200m Wetland	SHAPE_MN	(+)	(+)
	LSI	(-)	(-)
1200m Ag/Forest	SHAPE_MN	(+)	(+)
	LSI	(-)	(-)
1600m Forest	SHAPE_MN	(+)	X
1600m Wetland	SHAPE_MN	(+)	X
	LSI	(-)	X
1600m Ag/Forest	SHAPE_MN	(+)	(+)
	LSI	(-)	(-)
2000m Forest	PLADJ	(+)	X
2000m Wetland	PLADJ	(-)	(+)
2000m Ag/Forest	SHAPE_MN	(+)	(+)
	LSI	(-)	(-)

Results from the assessment of candidate model predictive performance using RMSPE are provided in Tables 3 and 4 illustrates actual versus predicted BU rates at 18 sample site locations. Here, the spatial 1200m wetland model, which included SHAPE_MN and LSI as predictor variables achieved the lowest RMSPE. Parameter estimates for this model are given in Table 5. Here, and as noted in Table 2, the regression coefficients associated with SHAPE_MN and LSI were both positive and statistically different from zero, which suggests that more complexly-shaped and aggregated wetland land cover patches surround villages with higher BU rates. The small value of the spatial decay parameter and hence the long effective spatial range (defined as the distance at which spatial correlation drops to below 0.05) suggests relatively strong residual dependence, even after accounting for the predictor variables.

**Table 3:** Root mean square predictive error of actual BU rates versus non-spatial and spatial GLM predicted rates per 1,000 people.

*RMSPE per 1*,*000 people*		
*Model*	*Non-spatial*	*Spatial*
800m Forest	3.0741	3.0708
800m Wetland	4.7621	4.7605
800m Ag/Forest	6.0901	6.0807
1200m Forest	2.5186	2.5083
1200m Wetland	1.8926	1.8711
1200mAg/Forest	4.2825	3.884
1600m Forest	5.8527	5.8507
1600m Wetland	2.3368	2.3353
1600m Ag/Forest	3.9576	3.9163
2000m Forest	1.8771	1.8764
2000m Wetland	2.2222	2.2181
2000m Ag/Forest	3.4825	3.2037

**Table 4:** BU actual rates vs. predicted rates per 1,000 people at 18 testing locations.

BU Rates per 1,000 People	
*Actual Rates*	*Predicted Rates*
0.12	1.73
0.00	2.16
1.04	1.17
0.00	0.74
2.04	1.33
1.04	0.16
0.00	0.94
6.69	2.25
0.75	3.01
1.93	1.25
2.35	0.24
0.00	1.38
0.00	0.21
0.19	1.28
0.00	0.46
0.00	1.26
0.00	1.67

**Table 5:** Credible intervals for 1200m wetland best-fitting spatial binomial GLM. Variables that maintain positive or negative signs across their corresponding interval region may be interpreted as having a high probability of the true value of the variable lying within this credible region.

*1200 m Wetland*			
	50%	2.50%	97.50%
(Intercept)	-8.1	-8.5	-7.2
SHAPE_MN	0.5	0.1	0.8
LSI	-0.2	-0.4	0
*σ* ^*2*^	4	3.6	4.3
*Φ*	0	0	0
Effective range	30.2 km	28.6 km	41.2 km
max intersite distance	172.4 km		

**Fig 5:**
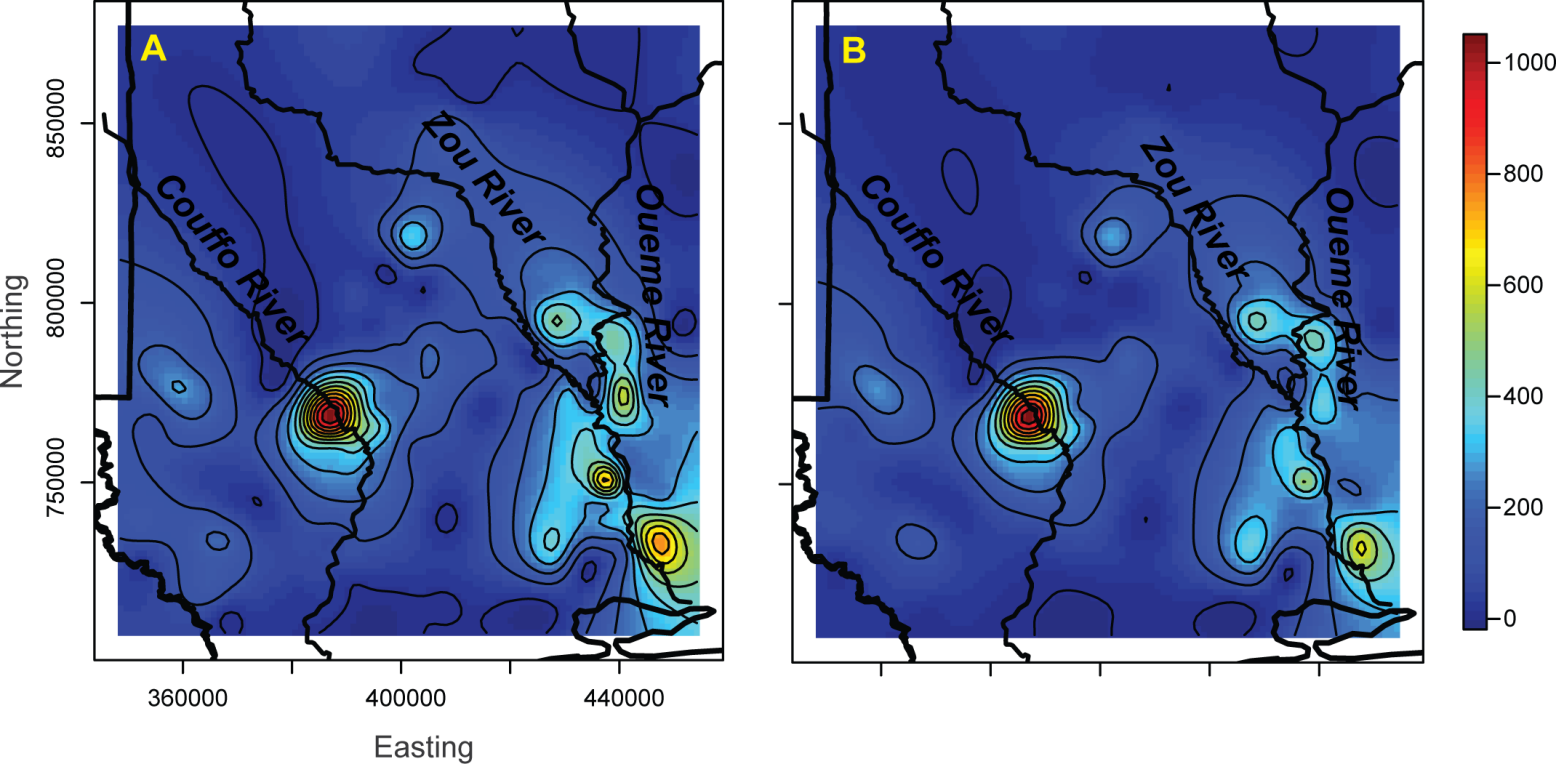
Observed (A) vs. fitted (B) BU rates.

**Fig 6:**
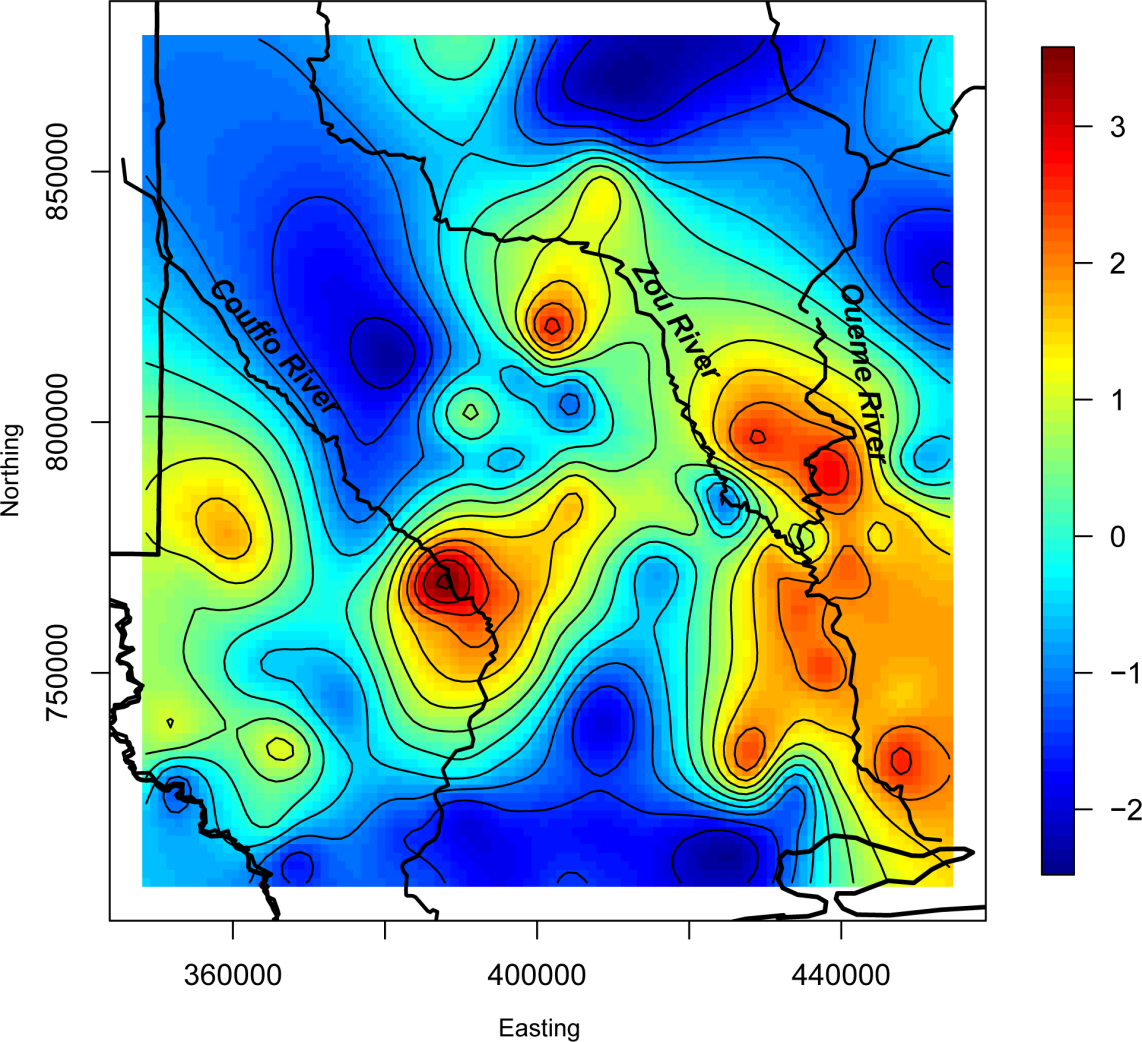
Map of spatial random effects. Red, orange, and yellow colors indicate areas where unknown, spatially structured variables that were not included in the model make a higher contribution to BU rates than in other areas of the study region.

**Fig 7:**
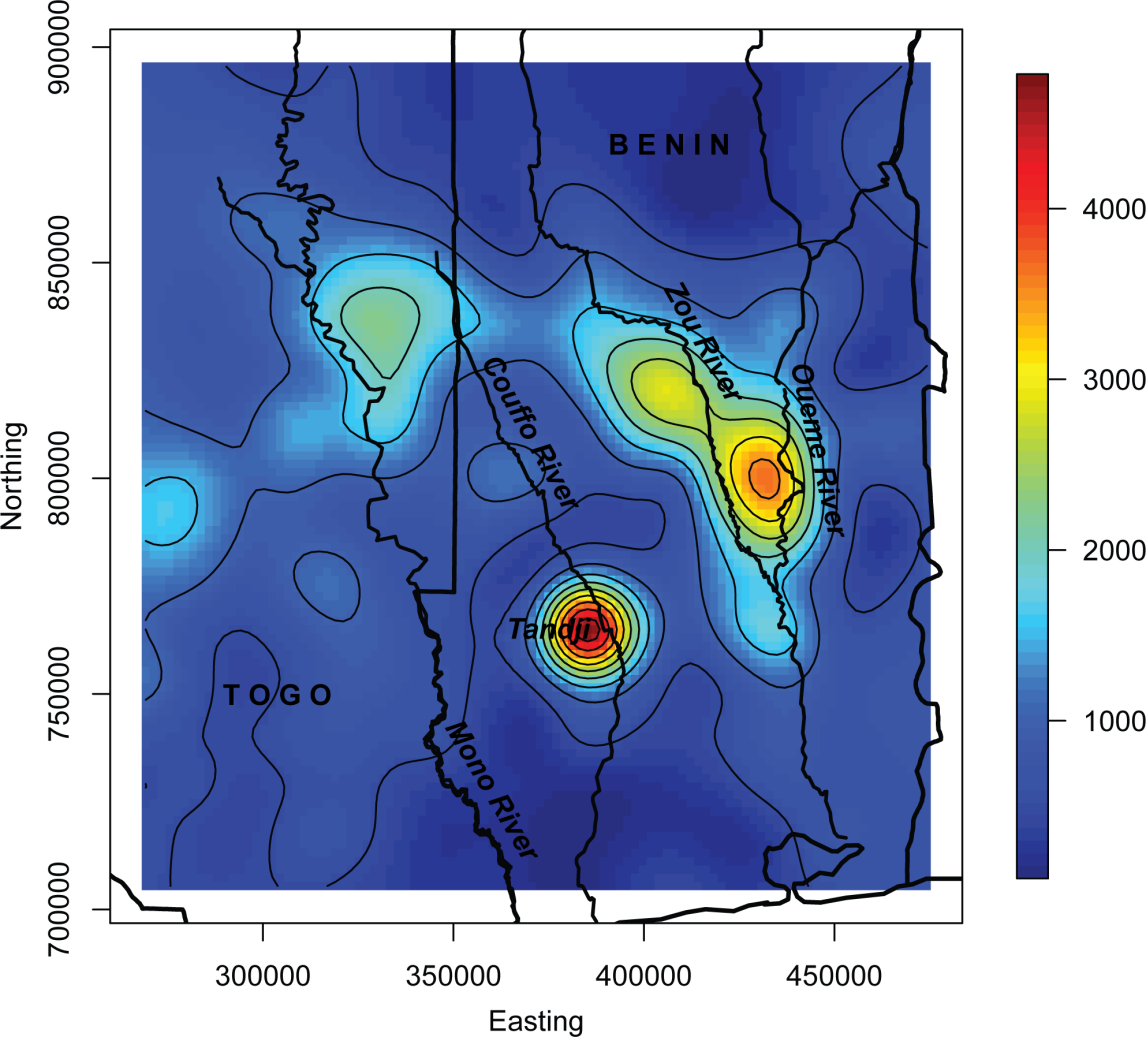
Interpolated mean of the posterior predictive distribution. Predicted rates per 100,000 individuals. Red, orange, and yellow colors indicate areas with higher BU rates predicted, while blue and green colors represent areas predicted to have low BU rates.

We examined plots of actual BU rates versus the 1200m wetland model predictions to assess spatial patterns in the model fit (Fig 5). There appeared to be relatively strong agreement in most areas, but BU prevalence rates were under-predicted along the Ouémé River. A plot of the spatial random effects highlighted areas where spatially structured predictor variables were missing from the model, influencing the spatial distribution of the rates and promoted model fit, with higher values near locations with case presence (Fig 6). An interpolated surface of the mean of the posterior predictive distribution at new locations based on the 1200m wetland model represents BU risk across the study region (Fig 7).

## Discussion

This study is the first to investigate land cover configurations indicative of anthropogenic disturbances in relation to BU incidence. Model results provided insight into BU incidence in West Africa, while demonstrating the value of spatial modeling approaches in disease ecology investigations. Lower DIC values corresponding to spatial models support the hypothesis that spatial structure exists for drivers of BU incidence in the region. Importantly, comparisons between non-spatial and spatial variable significance demonstrated the potential for inaccurate estimates to occur when using non-spatial models to address ecological problems. The inclusion of the spatial random effects accounted for missing predictor variables and provided substantial improvements in predictions of BU rates at unsampled locations (Table 2).

**Fig 8:**
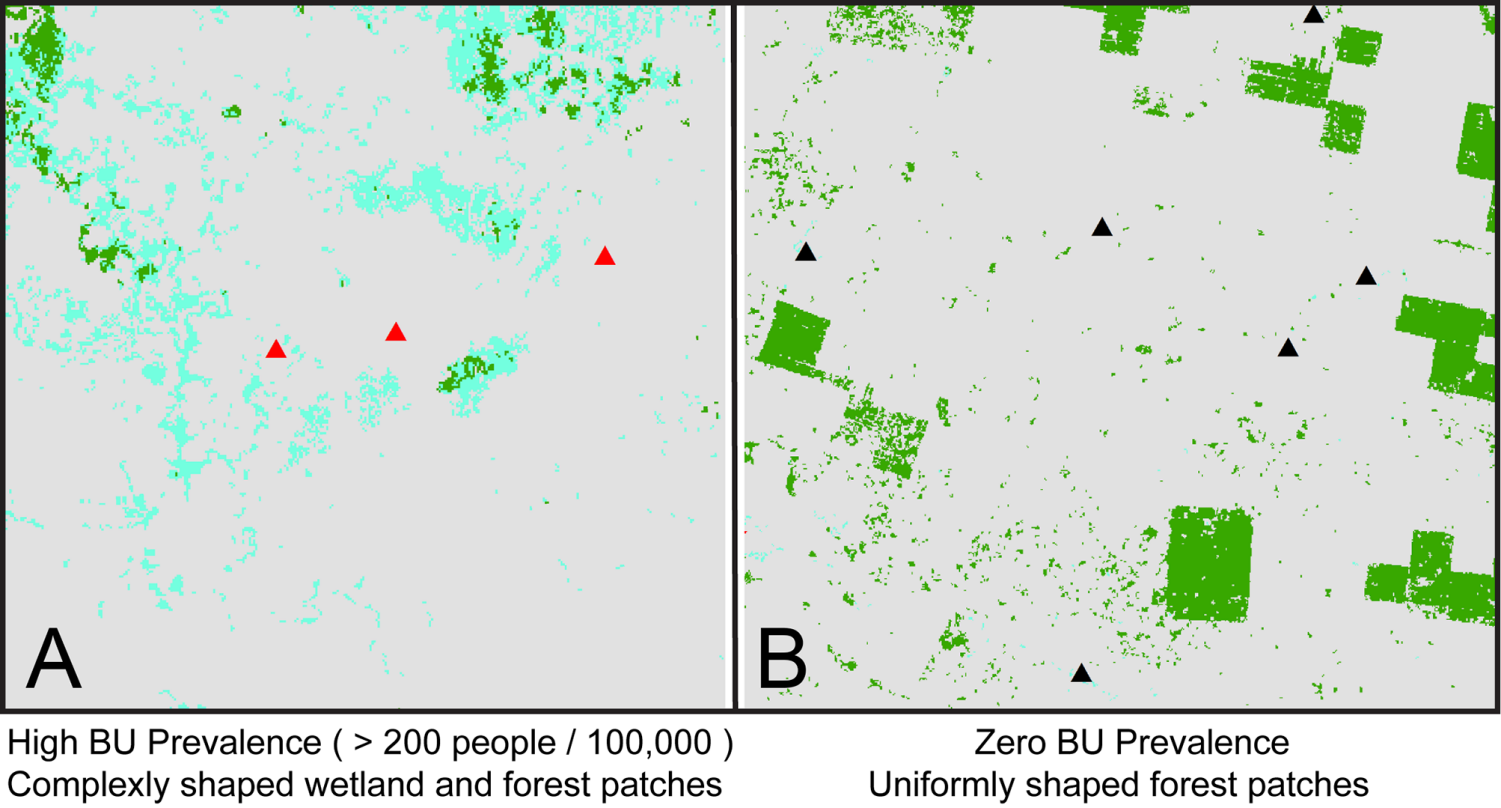
A) Areas with aggregated and complexly shaped wetland and forest patches surrounding locations with high BU prevalence. B) Uniformly shaped forest patches surrounding locations with no BU prevalence.

Our model results did not support the hypothesis that more uniformly-shaped and fragmented land-cover patches, indicative of potential anthropogenic disturbance, surround villages with higher BU rates. Spatial model results suggested general trends toward more aggregated and complexly-shaped, or natural, patches surrounding villages with higher BU rates, although inconsistencies in variable significance occurred across distance intervals. Fig 8 illustrates landscapes from our classification that represent high BU prevalence rates and no BU prevalence rates that correspond with our model results.

Mixed agriculture/forest class results suggested that more aggregated and complexly-shaped patches surrounded villages with higher BU rates at 1200m–2000m distances. While the configuration did not correspond to those recognized as representing anthropogenically-disturbed environments, the presence of a mixed agriculture and forest class suggests an inherent intrusion of agriculture into undisturbed forested landscapes. Persons tending fields in these mixed landscapes could have elevated opportunities to encounter edge effects and additional disturbances known to contribute to pathogen proliferation in environmental diseases [3]. Investigation into the role of ecological succession and specific disturbance stages in BU incidence may yield important information regarding high-risk areas.

Several plausible scenarios may explain model outcomes. The first considers the regions in which most landscape ecology studies identifying shapes indicative of anthropogenic disturbance took place. These studies occurred largely in developed regions of Europe and the United States, rather than in developing areas, such as Benin. Further investigation into quantification of land cover attributes in developing versus developed regions may provide useful information for future studies. Additionally, the models constructed in this study may not have been inclusive of land cover classes that could play an important role in BU ecology. Further investigation into additional land cover classes may yield insightful results.

Another consideration is the scale at which the investigation took place. This study utilized 30m resolution satellite imagery with a 5x5 statistical majority filter to characterize the landscape for statistical analyses. Drivers promoting BU proliferation may operate at coarser or finer scales. Additionally, several landscape disturbances associated with anthropogenic activities may not have been identified using medium-resolution imagery. One example is the presence of cultivated rice paddies within wetland land cover. Uniformly-shaped plots indicative of cultivation were easily discernible in higher-resolution 4m Ikonos imagery, but these disturbances were not visible in the Landsat imagery; thus, some areas that we treated as natural wetlands may have been anthropogenically impacted as well. Therefore, additional studies investigating land cover configurations at multiple scales could benefit BU research in the region.

Identification of spatial autocorrelation effects in model residuals supported the hypothesis that a spatial structure exists for drivers of BU incidence. The effective range of the “best” model suggested that spatial autocorrelation exists within 28.6km—41.2km of village centers and contributes to shaping the distribution of BU cases in the region. Although the spatial random effects do not indicate what is driving the presence of BU, the spatial patterns provide clues about missing variables and improved the predictive accuracy of the BU risk surface. Visual inspection of the spatial random effects reveals a pattern that corresponds roughly with a geologic and soil type transition from sedimentary basins with ferralitic and hydromorphic soils in southern Benin to a crystalline basement with fersialitic soils in the central region, warranting further exploration of these phenomena in relation to the spatial distribution of BU incidence in this region (IMPETUS Atlas Benin).

The purpose of the risk surface was to act as a preliminary guide to identifying regions where BU cases would be most likely to occur. Model results identified high-risk areas in known endemic regions near the town of Tandji and between the Zou and Ouémé Rivers in Benin, demonstrating consistency with case incidence reports [58]. Results also suggested high rates along the Ouémé River in the east and along the Couffu River in the western area of the country. Lower predicted rates along the southern coast could be the result of brackish waters that negatively impact environmental conditions suitable to the MU pathogen, or may be attributed to the hypothesis that residents in this region have better access to pumped water due to greater urbanization, which may lower BU risk [21]. Further, sandy soil, which is quick to drain and does not promote the accumulation of standing water, may hinder opportunities for MU growth in this region. Additionally, model results suggested lower rates between the Couffu and the Ouémé and Zou rivers, where higher elevations separate two known endemic regions, reiterating results from previous studies [21,22,59].

More interesting were predicted BU risk areas within the boundary of Togo. Model results suggested moderate risk east of the Mono River, but north of where the river delineates the international border. This region houses the Nangbeto Dam, behind which a large reservoir resides. Although this region is located within a wetland system, few BU cases were reported following construction of the Nangbeto dam in 1987 (R. Christian Johnson, personal communication, September 20, 2015). One hypothesis is that a reduction in cases occurred because of controlled fluctuations in water levels, reducing seasonal flooding impacts in the region, similar to the situation in Ghana, where few BU cases are known to occur in the Lake Volta region [11]. While the environmental conditions may be comparable to those identified as high risk environments in Benin, the model could not account for anthropogenic interference with river flow and therefore, predicted this area as having a moderate BU risk.

Generally, predicted BU rates in Togo exhibited moderate values with less spatial variability than those in Benin. This trend may result from the increased distance from observed locations. As distance from locations with known values exceeds the effective range, predicted rates have a tendency to move toward a mean predictive value. Although this phenomenon may have impacted predicted rates within Togo, identification of regions at moderate risk for BU occurrence is a first step in bridging knowledge gaps stemming from data disparities in the region.

### Conclusions

The role of anthropogenic ecosystem disturbances in the emergence of environmental bacterial infections is poorly understood. This study was a first attempt to link land cover configurations representative of anthropogenic disturbances to the environmental bacterial infection Buruli ulcer disease. Although results did not suggest a positive relationship between land cover patch configurations representative of anthropogenic disturbances and BU rates, study results identified several significant variables, including the presence of natural wetland areas, warranting future investigations into these factors at additional spatial and temporal scales.

Beyond the novel exploratory analysis outlined above, a major contribution of this study included the incorporation of a modeling component that partitioned the spatial structure of missing variables, providing a structure from which to predict BU rates to new locations without strong knowledge of environmental factors contributing to disease distribution at the beginning of this analysis. The resulting continuous BU risk surface demonstrates the potential to develop and to target surveillance efforts using spatial modeling approaches. The ability to predict potential risk adequately to locations where few data are available provided a first step toward prevention, while creating a tool from which a more systematic and controlled site selection process may be used to target future environmental sampling research. Continued acquisition of accurate, georeferenced case data along with georeferenced pathogen data will provide the best opportunity for robust empirical studies of relationships between ecological factors, anthropogenic activities, and BU transmission.
